# Region-based epigenetic clock design improves RRBS-based age prediction

**DOI:** 10.1111/acel.13866

**Published:** 2023-05-11

**Authors:** Daniel J. Simpson, Qian Zhao, Nelly N. Olova, Jan Dabrowski, Xiaoxiao Xie, Eric Latorre-Crespo, Tamir Chandra

**Affiliations:** MRC Human Genetics Unit, MRC Institute of Genetics and Cancer, University of Edinburgh, Edinburgh, UK

**Keywords:** aging, epigenetic age, epigenetic clocks, prediction, regional clocks, RRBS

## Abstract

Recent studies suggest that epigenetic rejuvenation can be achieved using drugs that mimic calorie restriction and techniques such as reprogramming-induced rejuvenation. To effectively test rejuvenation in vivo, mouse models are the safest alternative. However, we have found that the recent epigenetic clocks developed for mouse reduced-representation bisulphite sequencing (RRBS) data have significantly poor performance when applied to external datasets. We show that the sites captured and the coverage of key CpGs required for age prediction vary greatly between datasets, which likely contributes to the lack of transferability in RRBS clocks. To mitigate these coverage issues in RRBS-based age prediction, we present two novel design strategies that use average methylation over large regions rather than individual CpGs, whereby regions are defined by sliding windows (e.g. 5 kb), or density-based clustering of CpGs. We observe improved correlation and error in our regional blood clocks (RegBCs) compared to published individual-CpG-based techniques when applied to external datasets. The RegBCs are also more robust when applied to low coverage data and detect a negative age acceleration in mice undergoing calorie restriction. Our RegBCs offer a proof of principle that age prediction of RRBS datasets can be improved by accounting for multiple CpGs over a region, which negates the lack of read depth currently hindering individual-CpG-based approaches.

## 1 Introduction

DNA methylation-(DNAm)-based epigenetic clocks have become well-established as predictors of chronological age (Bocklandt et al., 2011; Hannum et al., 2013; Horvath, 2013; Koch & Wagner, 2011; Levine et al., 2018; Lu et al., 2019; Weidner et al., 2014; Zhang et al., 2019). The predicted age generated by these clocks is referred to as epigenetic age (eAge) and the age acceleration (difference between eAge and chronological age, chAge) is associated with a number of disease states, conditions and all-cause mortality (Chen et al., 2016; Fiorito et al., 2021; Higgins-Chen et al., 2020; Horvath, 2015; Horvath et al., 2014, 2016, 2018; Horvath & Levine, 2015; Jansen et al., 2021; Kim et al., 2022; Lu et al., 2019; Maierhofer et al., 2017; Marioni et al., 2015; Martin-Herranz et al., 2019; Simpkin et al., 2016; Wu et al., 2019). As a result, eAge has become a widely used proxy to measure biological age, that is, a measurement of age that may act as a better predictor of health and mortality than chronological age (Baker & Sprott, 1988; Simpson & Chandra, 2021).

**Figure 1:**
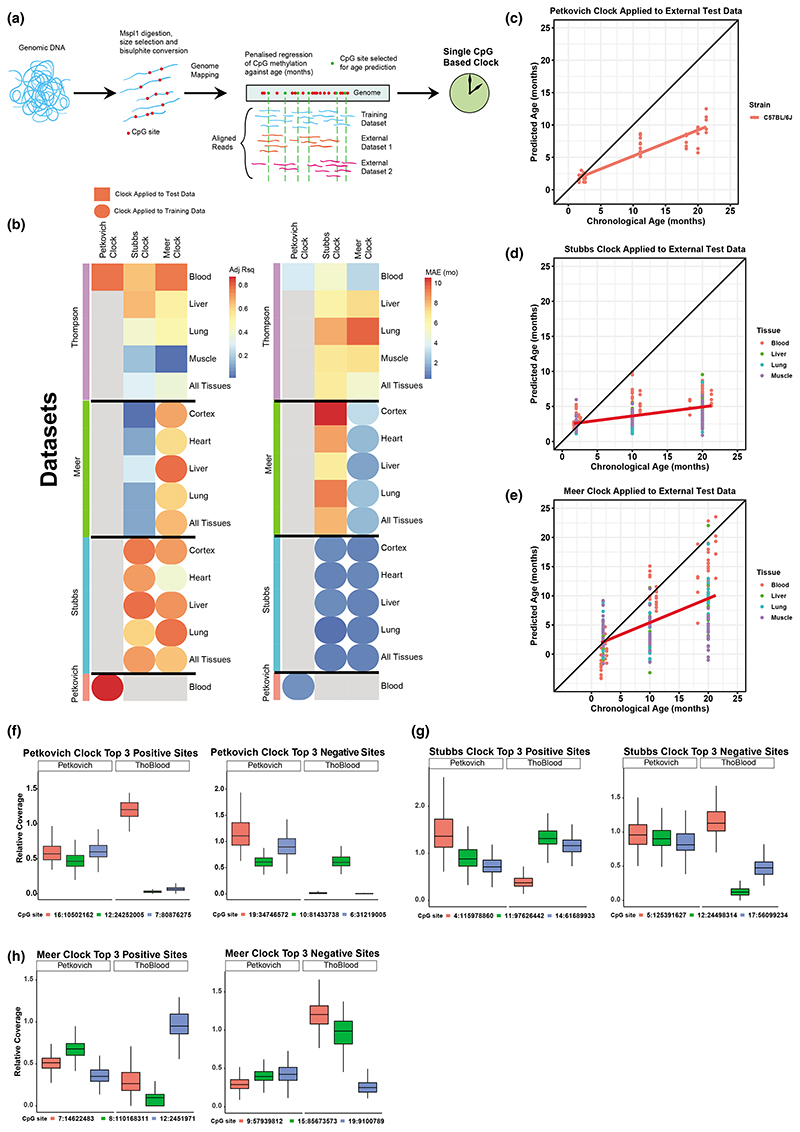
Mouse RRBS blood clocks are not as efficient when applied to external datasets (data independent of particular clock study). (a) Schematic of RRBS clock construction, with penalized regression conducted on methylation levels of single CpGs against age. (b) Adjusted *R*^2^ (left) and median absolute error (MAE, right) of various clocks applied to tissues from various datasets (Meer et al., 2018; Petkovich et al., 2017; Stubbs et al., 2017; Thompson et al., 2018). Round points represent clocks applied to training datasets, square points represent clocks applied to external datasets. (c–e) Petkovich (c), Stubbs (d) and Meer (e) mouse RRBS clocks applied to external Thompson multi-tissue dataset. (f, g) Relative coverage of the top three positive weighted sites (left) and top three negative weighted sites (right) of the Petkovich (f), Stubbs (g) and Meer (h) clocks in the Petkovich et al., 2017 and Thompson et al., 2018 datasets.

Most human epigenetic clocks are based on methylation arrays, which offer precise and reproducible readouts of thousands of CpGs. Epigenetic age prediction in model organisms such as mouse enables the quantification of lifespan and healthspan interventions (Simpson et al., 2021; Simpson & Chandra, 2021). However, until recently, methylation arrays were restricted to human, and while arrays have become available for other mammals (Arneson et al., 2022; Zhou et al., 2022), they do not cover non-mammalian model organisms such as zebrafish. Reduced-representation bisulphite sequencing (RRBS) offers an alternative species-agnostic approach for the development of epigenetic clocks. For example, a number of mouse epigenetic age-predictors have been created using RRBS (Meer et al., 2018; Petkovich et al., 2017; Stubbs et al., 2017; Thompson et al., 2018; Trapp et al., 2021; Wang et al., 2017; see Table S1 for a summary table of some of the main mouse RRBS clocks). In the original RRBS protocols, genomic DNA was enriched in CpG sites through enzymatic digestion with MspI (an enzyme that cuts DNA at CCGG sites, regardless of their methylation state; Baheti et al., 2016; Gu et al., 2011; Meissner et al., 2005) (Figure 1a). This process is a cheaper alternative to whole genome bisulphite sequencing (WGBS) since it captures only ~1% of the genome, achieving a higher relative depth of coverage (Gu et al., 2011; Meissner et al., 2008). However, RRBS datasets vary considerably in coverage and quality in comparison to the array format, which is highly standardized. This is possibly due to differences in library preparation protocols which could lead to inaccuracies when applying epigenetic clocks from one dataset to the next (Field et al., 2018; Simpson & Chandra, 2021; Thompson et al., 2018).

Here we show that the overlap of CpG sites captured in RRBS and their coverage differ between datasets, leading to poor transferability of clock algorithms between them. To overcome this limitation, we present a novel design strategy, generating clocks based not on individual CpG sites but on the average methylation level observed in contiguous regions. More precisely, we present two strategies, sliding windows and density-based clustering, to detect regions of interest that form the basis of our proposed regional clocks. These regional clocks outperform their individual-CpG-based counterparts both in accuracy and transferability. Furthermore, we show that they are more robust when applied to low-coverage, downsampled data and capture biological age.

## 2 Results

### 2.1 Uneven coverage contributes to RRBS clocks inaccuracy when applied to external datasets

We first analyzed the performance of published mouse RRBS clocks against training data and data external to the study used to construct a particular clock. As expected, the clocks originating from Petkovich et al., 2017; Stubbs et al., 2017; Meer et al., 2018 (hereafter referred to as Petkovich, Stubbs and Meer clocks, collectively referred to as individual-CpG-based clocks) had a high correlation and low error when applied to their training data (Figure 1b). When these clocks were applied to external Thompson et al., 2018 C57BL/6 multiple tissue RRBS data, their overall performance was poor; median absolute error (MAE) was high (range 2.9–9.6months) and *R*^2^ varied greatly (0.86–0.01) in age prediction (Figure 1c–e). The best performance was achieved by the Petkovich and Meer clocks applied to blood (with *R*^2^ correlations of 0.86 and 0.85, respectively); however, their errors were still comparatively high (MAE = 3.5 months for Petkovitch, MAE = 2.9 months for Meer) versus their training data (MAE = 1.2 months for both clocks).

We hypothesized that the performance of RRBS clocks on external datasets is due, at least in part, to uneven coverage of CpGs captured across datasets from different studies (Field et al., 2018; Simpson & Chandra, 2021; Thompson et al., 2018). To test whether uneven coverage contributes to the observed inaccuracies, we plotted from the Petkovich (used to train the Petkovich and Meer clocks) and Thompson datasets the relative coverage (normalized by total read count per sample) of the top three positive and negative weighted CpGs (as derived from their linear regression model) from the Petkovich, Stubbs and Meer clocks (Figure 1f–h). We show that many of the top weighted CpGs required for age prediction in a given clock had a coverage close to zero in the Thompson external dataset, which may contribute to RRBS-based clocks not working as effectively on external datasets.

### 2.2 Generating regional blood epigenetic clocks

**Figure 2:**
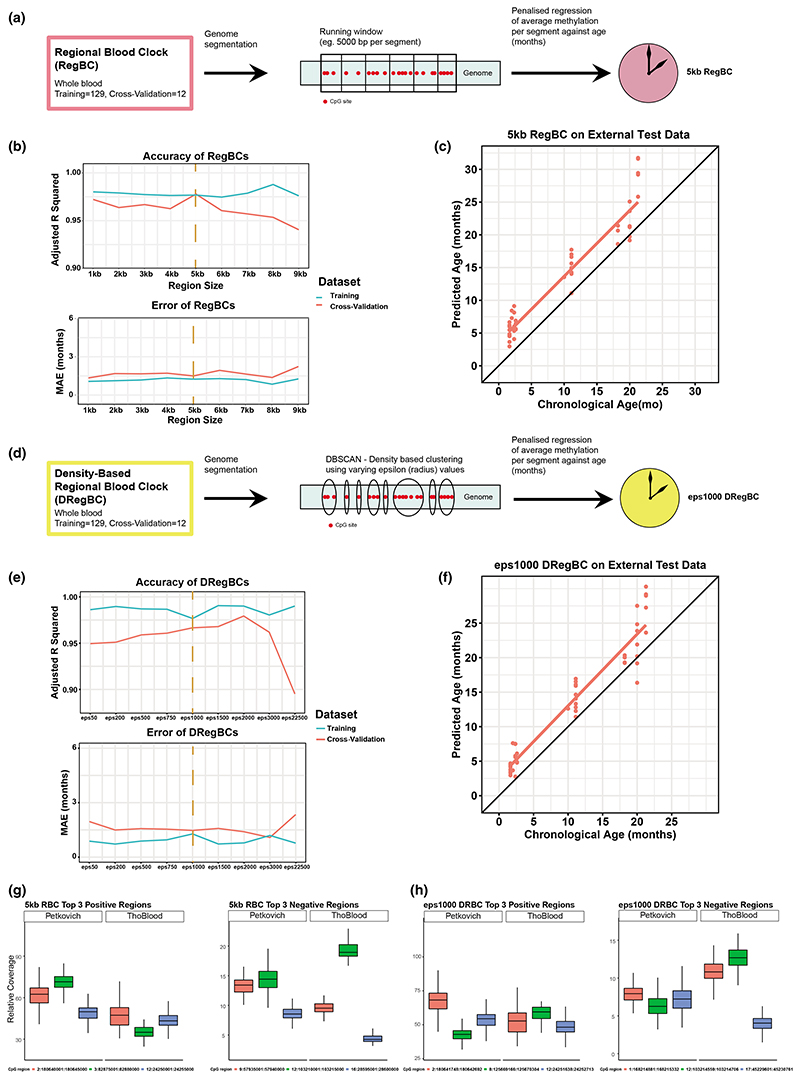
5 kb genome window size and density-based clustering provide transferable age prediction. (a) Graphical overview of regional blood clock (RegBC) creation. A running window generator was applied to segment the mouse genome into various sizes (eg. 5 kb). Segments for each particular window size were trained against age (months, mo) using penalized regression, resulting in a given clock for a particular window size. (b) Nine RegBCs were generated (1–9 kb region sizes). Correlation (Adjusted *R*^2^ of epigenetic age correlated with chronological age, top) and error (MAE, bottom) of each RegBC (*x*-axis) applied to a particular dataset. Blue and red lines are internal training and cross-validation datasets respectively (Petkovich et al., 2017). Orange vertical dashed line highlights the 5 kb RegBC as the best clock selected and taken forward. (c) 5 kb RegBC applied to Thompson et al., 2018 external dataset. (d) Graphical overview of density-based regional blood clock (DRegBC) creation. The density-based spatial clustering of applications with noise (DBSCAN) algorithm was applied to the CpGs in the Petkovich et al., 2017 blood training data to group them based on distance. Various epsilon (eps) values were used to govern the distance (in base pairs) between each CpG to form a cluster. For each eps value, the resulting clusters were then trained against age using penalized regression, resulting in a given clock. (e) Nine eps values varying from 50 to 22,500 were used to construct DRegBCs. Correlation (Adjusted *R*^2^ of epigenetic age correlated with chronological age, top) and error (MAE, bottom) of each DRegBC (*x*-axis) applied to a particular dataset (colours represent the same datasets as in b). Orange vertical dashed line highlights the eps1000 DRegBC as the best clock selected and taken forward. (f) eps1000 DRegBC applied to Thompson et al., 2018 external dataset. (g) Relative coverage of the top three positive weighted sites (left) and top three negative weighted sites (right) of the 5 kb RegBC in the Petkovich et al., 2017 training data and Thompson et al., 2018 external dataset. (h) Relative coverage of the top three positive weighted sites (left) and top three negative weighted sites (right) of the eps1000 DRegBC in the Petkovich et al., 2017 training data and Thompson et al., 2018 external dataset.

It has been previously shown that proximal CpGs show correlated methylation behavior (Lövkvist et al., 2016). We therefore explored whether taking the averaged CpG methylation value over a region (rather than from individual CpG positions) would overcome uneven coverage issues from RRBS data, resulting in more transferable age prediction (Figure 2a). We used the mouse C57BL/6 whole blood training data from the Petkovich blood clock (aged 3–35months, training = 129, cross-validation = 12, sites with less than 5 reads filtered out; Petkovich et al., 2017) and segmented the mouse genome into various region sizes (1–9 kb) using sliding windows. For each window size, we calculated the averaged methylation levels from individual CpG sites within each window and conducted LASSO-penalized regression against chronological age to select optimal regions and weights for age prediction. Nine regional blood clocks (RegBCs) were generated (1–9 kb region sizes each, see Table S3 for all RegBCs applied to training and cross-validation datasets). Considering *R*^2^ and MAE, we observed that the RegBCs' performance on training and cross-validation data is relatively constant up to 6 kb window size, after which the *R*^2^ in the cross-validation data decreases (Figure 2b). Irrespective of the particular choice of window size, all RegBCs generated had *R*^2^ values higher than 0.93 and a MAE lower than 3 months when applied to training or cross-validation data, showing that regional clocks can perform at least as well as individual-CpG-value-based predictors. To investigate the extended power of regional clocks, we then proceeded to test the transferability of this new model to unseen data. For this we chose to test on an external blood dataset (Thompson et al., 2018) the 5 kb window regional blood clock (5 kb RegBC) as it has the smallest difference in error and *R*^2^ between the training and cross-validation dataset. The 5 kb RegBC showed a higher correlation and lower error (*R*^2^ = 0.91, MAE = 3.38, Figure 2c) than the blood-specific Petkovich clock trained on individual CpGs (*R*^2^ = 0.86, MAE = 3.52, Figure 1c). The 5 kb RegBC also has a higher *R*^2^ than the Meer clock applied on the Thompson data, albeit the Meer clock having a lower MAE (*R*^2^ = 0.85, MAE = 2.94, Figure 1e). The 5 kb RegBC has 24 negatively weighted regions (regions that hypomethylate with age) and 11 positively weighted regions (regions that hypermethylate with age) (Table S4). A total of 1978 CpGs are present in the 35 total regions of the 5 kb RegBC, with a mean of 57 CpGs per region.

Although the sliding window approach is widely used for whole genome segmentation, groups of CpGs with correlated methylation values could easily get separated into different regions, which may hinder age prediction. Therefore, we tested whether RegBCs built using CpGs clustered based on proximity to each other would improve age prediction. For this, we applied the density-based spatial clustering of applications with noise (DBSCAN) algorithm to the CpGs covered in the Petkovich RRBS blood training data (sites with fewer than 5 reads were filtered out) (Ester et al., 1996). To find clusters, we used the distance (base pairs) between CpG sites and required at least 5 CpG sites to form a cluster or region. DBSCAN then discovers regions of clustered CpG sites that are separated by a minimum distance or radius (epsilon value, eps), see Figure 2d. We created regions of CpGs using varying eps values between 50 and 22,500 bp. For each eps value, we again conducted LASSO-penalized regression against chronological age to select the optimal regions and weights to predict age. Nine density-based regional blood clocks (DRegBCs) were constructed with varying eps values (Table S3). All DRegBCs had *R*^2^ values exceeding 0.9 and an MAE less than 3 months when applied to the training and cross-validation datasets (Figure 2e). The eps1000 DRegBC had the smallest difference between training and cross-validation datasets in terms of both *R*^2^ and MAE (with the exception of the eps2000 DRegBC which had a lower MAE on its cross-validation test). We applied it to the external Thompson blood dataset, where it outperformed the Petkovich and Meer clocks based on individual CpGs (*R*^2^ = 0.92, MAE = 2.71, Figure 2f). The mean region size found in the eps1000 DRegBC is 623 bp, with a mean of 55 CpGs present per region. There are 33 regions in total, with 21 negatively weighted and 12 positively weighted.

To test whether our approach reduced coverage differences, we plotted the coverage for top weighted CpGs between data sets. We found that the relative coverage of the top three hyper/hypomethylating regions in the 5 kb RegBC and eps1000 DRegBC in the Petkovich and Thompson datasets is largely maintained using the regional approach (Figure 2i,j). Hence, taking the averaged value of CpG methylation over regions appears to improve relative proportions of reads for clock components between datasets compared to individual CpG approaches (Figure 1f–h), which may contribute to the improved age prediction in external data sets.

Between the 5 kb RegBC and eps1000 DRegBC, 21 5 kb RegBC regions overlap with 22 eps1000 DRegBC regions (with one DRegBC site overlapping with two RegBC windows) (Table S4). These overlapping regions (many of which were top weighted in both clocks) were also associated with various genes (*Tcfl5*, *Map10*, *Smarca5-ps*, *Rbm46*, *Aldh1a2* and *Gm21297* in hypermethylating regions and *Prima1*, *Fgf12*, *Eml4*, *Qrfp*, *Hpn*, *Prr13* and *Ntn1* in hypomethylating regions). We also found a number of CpGs from the Petkovich and Meer clocks present in our 5 kb RegBC and eps1000 DRegBC regions, as expected from these clocks being created using the same training data. 16 Petkovich clock CpGs overlap with 6 regions of 5 kb RegBC, while 15 overlap with 5 regions of the eps1000 DRegBC, all of which are regions shared by both clocks except one site/region unique to the 5 kb clock. The Meer clock is more represented in both clocks, with 23 CpGs in 7 of the 5 kb RegBC regions, while 19 Meer clock CpGs are overlapping with 5 eps1000 DRegBC regions, all of which are shared with the 5 kb RegBC. 11 Meer CpG sites were present in one particular region in the eps1000 DRegBC, 2:164167686:164169038, which is also in a 5 kb RegBC region. This suggests that, as expected, the eps1000 DRegBC finds more targeted regions than the 5 kb RegBC clock.

### 2.3 Biological topography and relevance of regional blood clocks

**Figure 3:**
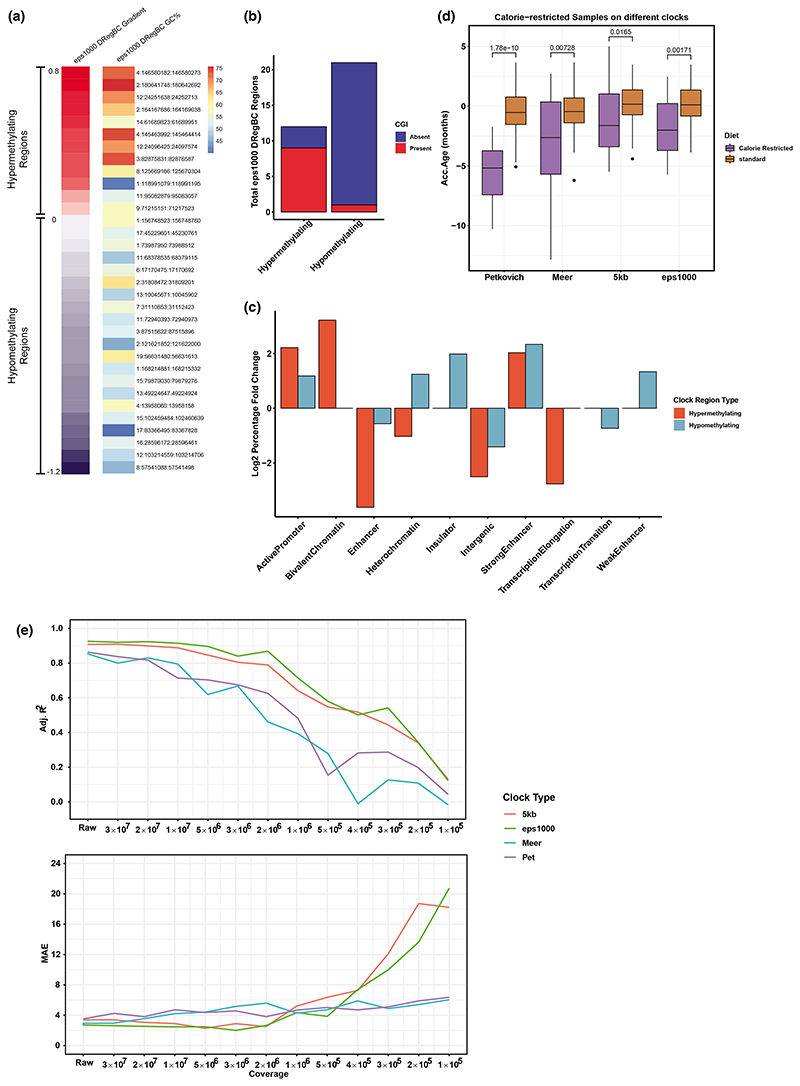
Chromatin structure and biological age prediction of eps1000 DRegBC (a) Percentage GC content of eps1000 DRegBC regions ordered by the gradient of methylation change with age. (b) Number of eps1000 DRegBC regions overlapping with CpG islands (CGIs) in hypermethylating and hypomethylating regions. (c) Log2 fold change of ChromHMM states proportions in eps1000 DRegBC hypermethylating or hypomethylating regions compared to RRBS genomic proportions captured in Petkovich 2017 training data. (d) Petkovich, Meer, 5 kb RegBC and eps1000 DRegBC applied to calorie restricted and control samples. (e) Correlation (adjusted *R*^2^ of epigenetic age correlated with chronological age, top) and error (MAE, bottom) of 5 kb RegBC, eps1000 DRegBC, Petkovich and Meer clocks applied to downsampled Thompson et al., 2018 external dataset.

There is a large degree of overlap between the 5 kb RegBC and the eps1000 DRegBC, however, regions captured by the eps1000 DRegBC are on average smaller. This would suggest the DBSCAN method identifies more discrete regions that might allow us to capture some degree of underlying biological relevance. We first plotted the genomic GC content percentage for each region, ordered by the slope of methylation change with age. Globally, AT-rich regions tend to be heterochromatic and constitutive lamina-associated domains (Bolzer et al., 2005; Chandra et al., 2015; Federico et al., 2004, 2006; Gilbert et al., 2004; Meuleman et al., 2013; Saccone et al., 2002). We observed a strong correlation (*R* = 0.70) between average GC content and hypo/hypermethylation gradient (Figure 3a,b). Hypermethylating regions show an average GC content as high as 80%, while hypomethylating regions were as low as 40% (Figure 3a). This suggests that age-dependent hypomethylation is associated with heterochromatic areas, consistent with previous observations (Bollati et al., 2009). In contrast, CpG islands (CGIs) were almost exclusively found in hypermethylating regions (Figure 3b), consistent with previous studies that show CGIs tend to gain methylation with age (Christensen et al., 2009; Gopalan et al., 2017; Heyn et al., 2012; Johansson et al., 2013; Slieker et al., 2018).

To annotate the eps1000 regions in more detail, we looked at their overlap with ChromHMM chromatin states (Figure 3c; see Table S5) (Ernst & Kellis, 2012; Pintacuda et al., 2017). We calculated the percentage bp of each ChromHMM state found within each eps1000 DRegBC window and calculated the log2 fold difference between chromatin proportions in the clock regions and the whole RRBS genome captured in the Petkovich et al., 2017 training data (see in Section 4.9). Compared to the genome, the eps1000 DRegBC regions had a lower proportion of intergenic regions and a higher proportion of active promoters. The eps1000 DRegBC had a higher proportion of heterochromatin in hypomethylating regions compared to hypermethylating regions, which is consistent with the GC proportions in Figure 3b and previous findings (Bollati et al., 2009). Bivalent chromatin occurs at a higher proportion in hypermethylating than hypomethylating regions, which is consistent with hypermethylation of bivalent chromatin as a hallmark of aging and with previous clock studies (Horvath, 2013; Rakyan et al., 2010).

### 2.4 Regional blood clocks capture biological age

To investigate whether the 5 kb RegBC and eps1000 DRegBC can capture biological age, we applied each clock, as well as the Petkovich and Meer clocks, to calorie restricted (CR) samples (Figure 3d). The CR samples were aged 10, 18, 23, 27 months, were from the same dataset as the Petkovich training dataset (not included in training the RegBCs), and all had dietary intervention starting at 14 weeks old (Petkovich et al., 2017). The control group (*n* = 74) were C57 mice aged 10–28 months sampled from the Petkovich blood data. CR samples showed a significant age deceleration (*p* < 0.05) for both 5 kb RegBC and eps1000 DRegBC (median error = –1.6 and − 2.0 months, respectively). The Petkovich clock showed a greater age deceleration of −5.1 months, while the Meer clock has an age deceleration of −2.6 months, similar to the eps1000 DRegBC.

### 2.5 Regional blood clocks are more robust when applied to low coverage data

We next explored whether low coverage data could benefit from regional clock designs resulting in more robust age-prediction upon downsampling. We step-wise randomly downsampled the Thompson blood external dataset from raw (40 million reads) to 100,000 reads, and applied the 5 kb RegBC, eps1000 DRegBC, Petkovich and Meer clocks to each level of downsampling (Figure 3e). Both regional-based clocks maintain an *R*^2^ above 0.75 up to 2 million reads, while at this point, the Petkovich clock declined in correlation to ~0.46. Both RegBCs have an MAE lower than 4 months until 1 million reads. When coverage is reduced past 500,000 reads, the RegBCs still maintain a higher *R*^2^ than the Petkovich and Meer clocks. However, error increases rapidly for both RegBCs, while the Petkovich and Meer clocks maintain an error of ~5 months. We hypothesized that this difference in error might be due to a desaturation of CpG sites with downsampling, whereby as the total coverage gets closer to zero, the MAE for a given clock will eventually reach a limit value governed by the Y-intercept of each clock. For example, the intercept of the Petkovich and Meer clocks is 8.72 and 7.71 respectively, while the 5 kb RegBC and eps1000 DRegBC are 32.35 and 34.16, respectively. Hence, when downsampling results in a particular number of zero methylation sites, the Petkovich and Meer clocks appear to have a low error, which is a manifestation of how the clocks were built. To investigate this, we looked at the number of CpGs/regions with a methylation value of zero at each downsampling step. Indeed, the number of zero CpG sites/regions steadily increases for all clocks as coverage decreases (Figure S1). After downsampling past 1 million reads, the number of zero CpG sites start to saturate the total number of sites in the Petkovich and Meer clocks (Figure S1A,B). The RegBCs, by comparison, maintain a lower number of zero regions and do not reach saturation even down to 100,000 reads (Figure S1C,D). Hence, the regional-based clocks maintain a stronger age correlation with downsampling than individual-CpG-based clocks as it is harder for large regions to desaturate. However, since error starts to increase below 2 million reads in our regional clocks, we would not recommend a coverage value lower than this in RRBS experiments. In addition, we noticed both individual-CpG-based clocks (particularly the Petkovich clock) have a higher number of zero methylation CpG sites than zero methylation regions in the RegBCs for the raw Thompson dataset (Figure S1) which is consistent with similar observations in Figure 1f–h.

## 3 Discussion

Age prediction utilizing RRBS data has proven difficult due to the uneven coverage of CpG sites captured between different experiments. We have shown that a regional approach, based either on set window sizes or dynamically allocating windows based on CpG density, can improve the transferability of the age predictor when applied to external datasets as well as robustness when applied to low coverage data. Using samples from the same dataset used to create the Petkovich blood clock, our RegBCs outperform individual-CpG-valuebased clocks (Petkovich and Meer clocks) when tested on unseen external blood datasets. However, the main limitation of this study is the limited number of suitable RRBS datasets available for training and testing our approach.

An important characteristic of epigenetic age predictors is their ability to capture biological perturbations of aging. The regional blood clocks were able to detect a negative age acceleration in calorie-restricted mice. Our method was also able to offer more biological context by revealing the chromatin topography of regions useful for age prediction. Many regions were found to be shared by both the 5 kb RegBC and eps1000 DRegBC, with CpGs from published clocks also present. In these regions shared by both clocks, it is likely that the age-related methylation changes fall in the eps1000 DRegBC regions more specifically, since all the window sizes for the eps1000 DRegBC are smaller than the 5 kb windows. Indeed, one eps1000 DRegBC region was present in two 5 kb RegBC regions; hence, a cluster of CpGs with correlated methylation values was split up due to the sliding window approach, which may inhibit age prediction and biological interpretation. In addition, the eps1000 DRegBC was more accurate in predicting age than the 5 kb RegBC. Therefore, a cluster-based approach for assembling regions rather than a windowed approach might be more effective for both predicting age and gleaning biologically significant results. For example, a high proportion of bivalent (poised) chromatin was found in regions that hypermethylate with age in the eps1000 DRegBC, which is a known hallmark of aging (Rakyan et al., 2010). We also found enrichment of heterochromatin in hypomethylating regions, which is consistent with previous studies (Bollati et al., 2009). In addition, the majority of hypermethylating regions in our regional clocks were overlapping with CpG islands. This is, however, expected as CpG islands are commonly hypomethylated and, therefore, can only gain in methylation with age, which is a well documented phenomenon (Gopalan et al., 2017; Heyn et al., 2012; Johansson et al., 2013; Slieker et al., 2018). Indeed, CpG islands bound by PRC2 de novo methylate with age in certain tissue types (Klutstein et al., 2017; Maegawa et al., 2010) and methylation gain in these regions can be used to track aging (Moqri et al., 2022). It is therefore possible that the regions found in our clock may be similar regions where methylation is dysregulated with age; however, mechanistic studies are required to confirm any causal role in aging (Moqri et al., 2022).

Targeted methods have been developed for mice to reduce the cost that comes with RRBS and WGBS. Recently, the Wagner lab developed a 3 CpG and a 15 CpG clock for multiple platforms (pyrosequencing, droplet digital PCR and barcoded bisulphite amplicon sequencing), which offers accurate age prediction at few CpGs for relatively low cost (Han et al., 2018, 2020). Techniques such as this offer certain benefits for age prediction since they have a higher coverage at a small selection of CpGs, whereas RRBS experiments vary in coverage necessary for the CpG-specific approach for age prediction (Figure 1e,f). Our regional clocks offer a proof of principle that RRBS-based age prediction can be made more effective by accounting for multiple CpGs over a region and negating the lack of read depth at key CpGs that would otherwise hinder individual-CpG-based clocks. We show that regional clocks outperformed individual-CpG-based clocks when applied to downsampled datasets, showing that the averaged methylation over regions is able to compensate for low coverage. This offers a method to generate clocks that can be applied to sequencing data as shallow as 2 million reads. This principle could also be applied to RRBS datasets (and possibly WGBS) from other model organisms to develop age predictors. In addition, our technique has highlighted key regions that could be specifically targeted for accurate age prediction in mouse blood.

## 4 Methods

### 4.1 Data collection

The following mouse RRBS datasets were downloaded from the NCBI Gene Expression Omnibus (GEO); GSE80672 (141 C57 and 20 calorie-restricted C57 mouse; Petkovich et al., 2017), GSE120137 (*n* = 548; Thompson et al., 2018), GSE93957 (*n* = 62; Stubbs et al., 2017), and GSE121141 (*n* = 81; Meer et al., 2018).

The annotation information (metadata) of the sequencing samples, such as age and tissue, was downloaded from the NCBI SRA Run Selector. The unit of the chronological age is different among datasets, including month and week. For the convenience of subsequent calculations, all ages in weeks were converted to age in months. The conversion formula is:

(1) Age(mo)=Age(wk)×7÷30

where *Age*(*mo*) is an age in months, *Age*(*wk*) is an age in weeks, multiplied by 7 days per week and divided by an average of 30 days per month.

### 4.2 Data processing

The following processes were implemented via bash scripts on Eddie, a high performance computing cluster with a Linux-based operating system provided by the University of Edinburgh.

Read adaptors were removed by TrimGalore (version 0.5.0, – rrbs) which combines FastQC (version 0.11.4) and cutadapt (version 1.9.1; Krueger, 2012). The following processing steps were conducted with Bismark (version 0.18.1), a utility designed to process and map BS-seq data using packages such as bowtie2 (mapping, version 2.2.6) and samtools (read processing, version 1.6; Krueger & Andrews, 2011):

1. A bisulphite converted version of GRCm38.p6 reference genome was created by the Bismark genome preparation module (Krueger, 2016).
2. The trimmed reads were aligned to the bisulphite converted genome with Bismark (settings: –multicore 2 –phred33-quals -N 0-L 20).
3. Certain samples in the Petkovich dataset were processed twice on different flow cells. Duplicate sample BAM files were merged post mapping with Rsamtools (version 1.36.1; Morgan et al., 2022).
4. The Bismark methylation extractor module then obtained from the BAM files (generated in the second step) the mapped reads and methylation count information (CpG, CHG and CHH contexts). CHG and CHH sites were removed.
5. The bismark2bedGraph module was used to extract the methylation information from CpG context files and convert them to cov files (bismark.cov.gz) which contain six columns: (1) chromosome number; (2) start position; (3) end position; (4) percentage of methylated reads in total reads (β score); (5) the number of methylated reads; (6) the number of unmethylated reads (Krueger, 2016).

### 4.3 Coverage assessment

To compare whether there was a difference in coverage between all downloaded datasets (see Section 4.1 for list of datasets), the number of reads from the cov files was extracted using the “Read Count Quantitation” pipeline in SeqMonk (version 1.48.0; Andrews, 2007), as per the following steps:

1. Create a new project. “File” → “New project” → select genome GRCm38 v100 → click “Start New Project”.
2. Load RRBS data. “File” → “Import Data” → select “Bismark(Cov)” → select cov files in the new window.
3. Add annotation (for eps clock).“File” → “Import Annotation” → sele ct“Text(Generic)”
4. Define a probe.
  - For kb clock, “Data” → “Define Probes” → select “Running Window Generator” → set probe size.
  - For eps clock, “Data” → “Define Probes” → select “Feature Probe Generator” → select the annotation imported by the previous step (do not select “remove exact duplicate”).
  - For individual-CpG-based clocks, “Data” → “Define Probes” → select “Read Position Probe Generator” → select samples.
5. Define quantitation:
  - For coverage, “Data” → “Quantitate Existing Probes” → select “Read Count Quantitation” (do not choose “Correct for total read count” and “Log-Transform Count”).
  - For methylation value, “Data” → “Quantitation Pipelines” → ”Bisulphite methylation over features” → ”Run Pipeline”
6. Generate reports which contain coverage or methylation value. “Reports” → “Annotated Probe Report”

The coverage of Petkovich, Stubbs, Meer and Thompson blood data was extracted and organized into a matrix, which was loaded into R (version 4.1.1). The relative coverage was calculated as follows:

(2) $$RelativeCoverage\;=\;reads{\;}_{site\;}÷\;reads{\;}_{sample\;}\times 10^{6}$$

Representative sites (top three negatively and positively related to age) were selected based on their weight in a given clock. Each column represented a CpG site (or region of CpGs in the case of the regional clocks). Coverage of clock sites/regions in different datasets was plotted using ggplot2 (version 3.3.3; Wickham, 2016) in R (Figure 1f-h, Figure 2g,h).

### 4.4 Applying published BS-seq clocks to various datasets

The Petkovich blood clock and two multi-tissue (Meer and Stubbs) mouse BS-seq clocks were tested on various datasets. For each clock, the required CpG sites were extracted from a given dataset that the particular clock would be applied to (Table S1). Each clock was ran according to scripts and instructions from their respective publications, e.g. the to Run_Imputation. An R script provided by Stubbs et al. (2017) was used to run their clock (Meer et al., 2018; Petkovich et al., 2017; Stubbs et al., 2017; Thompson et al., 2018; Wang et al., 2017).

To evaluate the results, the adjusted R squared (adj. *R*^2^) and median absolute error (MAE) were calculated, and linear regression lines were illustrated with ggplot2 in R (Wickham, 2016). The formula for adj. *R*^2^ is as follows:

(3) $$\mathrm{Adj}\text{. }R^{2}=1-\left(1-R^{2}\right)((N-1))/((N-k-1))$$

where *N* represents the number of samples, and *k* represents the number of predictors (Miles, 2014). MAE is the median absolute error between epigenetic age and the chronological age, where if a test dataset has an MAE of X, then the age acceleration will differ by less than X in 50% of the samples (Horvath, 2013). The formula is as follows:

(4) MAE = median (|predicted age − chronological age|)

### 4.5 Genome segmentation

Before constructing a regional epigenetic clock, we filtered out any CpGs with fewer than five reads. Genome segmentation with set window sizes (e.g. 5 kb) was conducted using SeqMonk (version 1.47.0; Andrews, 2007), an interactive desktop application with a suite of tools for analyzing mapped genomic data. GRCm38 v100 was selected as the reference genome. Next, the filtered cov files were imported into SeqMonk, “Define Probe” was opened from the drop-down window of “Data”, and then “Running Windows Generator” was selected to set the window size (probe size) and step size. Window size and step size were set as equivalent, e.g. 5 kb for both to avoid any overlapping windows. After setting the probe, “Bisulphite methylation over features” was selected from “Quantitation Pipelines” to calculate the average methylation level per window/region. The formula of the average methylation level per region is:

(5) $$Regional\;methylation\;=\left(\sum^{\text{​}}CpG\;site\;methylation\;\right)/\left(\sum^{\text{​}}CpG\;sites\;\right)$$

where *CpG site methylation* is the mean methylation of an individual CpG site within a given region. This method was preferred than to take mean methylated reads and dividing by total unmethylated reads in a given region as this does not account for coverage bias due to a potential small number of highly methylated positions (Olova et al., 2018). There could also be very few reads, which would also result in an unreliable mean methylation call (Andrews, 2018). The results were exported as a counts matrix text file, where samples are columns and rows are genomic regions. Each dataset (see Section 4: Subsection 4.1) was segmented for all window sizes (1-9 kb) and were used for either training or testing the regional clocks.

For construction of the density-based RegBCs (DRegBCs), the locations of single CpG sites in the Petkovich training data was extracted using the “Read Position Probe Generator” function in SeqMonk to create probes for single CpGs. The coordinates were loaded into R and a custom for loop applied the dbscan function (from the dbscan package (Hahsler et al., 2019)) for various epsilon (eps) values (50, 200, 500, 1000, 3000, 22,500) to each chromosome. In the case of constructing a regional clock, the eps value is the maximum number of base pairs (distance) between each point to form a cluster. Minimum points to call a cluster was left at default (five points, i.e. 5 CpGs). The mean methylation for each cluster was calculated in SeqMonk in the same manner as the RegBCs above.

### 4.6 Regional epigenetic clock construction using LASSO

Each text file of region counts per region clock iteration (generated by SeqMonk) was loaded into R. All percentage methylation values for each region were divided by 100, so that 0–1 would represent 0%–100% respectively. Regions with NaN (i.e. no reads recorded) were removed. Least Absolute Shrinkage and Selection.

Operator (LASSO) regression model from the glmnet package in R (version 4.1.1) (Friedman et al., 2010) was applied. LASSO is a penalized regression model which selects the best CpG regions that correlate with age in a linear regression model:

(6) $$eAge=I+\beta_{1}X_{1}+\beta_{2}X_{2}+\ldots +\beta_{m}X_{m}$$

where *eAge* represents the epigenetic age, *I* is the intercept value where the linear model would meet the y-axis, *β* represents the average methylation score of a particular genomic region and *X* is the coefficient/weight (Hepp et al., 2016). We ran a 100-fold cross validation (using the cv.glmnet function) for each clock, saved the lowest lambda value determined and used this value when running the LASSO model for each clock (using the glmnet function; other than lambda, all other terms were default). A list of regions, their coefficients, and an intercept value were then generated.

Nine regional blood clocks (RegBCs, (1–9 kb region sizes) and nine density-based RegBCs (DRegBCs, varying eps values) were generated (Table S3). Whole blood from GSE80672 (training = 129, cross-validation test = 12; Petkovich et al., 2017) was used to construct the RegBCs. The Petkovich blood training dataset was randomly partitioned into 90% training and 10% cross-validation using the caret package (version 6.0–86; Kuhn, 2020) in R.

Each clock was applied to a particular dataset using a for loop written in R. It extracts each required CpG region (generated from LASSO penalized regression) from each sample in the counts matrix (generated from SeqMonk) and multiplies it with its corresponding weight (generated from the LASSO penalized regression) as according to Equation (6). Each iteration of the RegBC was applied to the blood training and cross-validation datasets (adj. *R*^2^ values were plotted in Figure 2b, left panel) and the Thompson et al. (2018) blood samples (GSE120137) (adj. *R*^2^ and MAE values were recorded in Table S3). Any values that were missing in the cross-validation or external datasets were imputed as zero.

### 4.7 Regional epigenetic clock applied to calorie restricted samples

RRBS data from calorie restricted C57 mice (CR, *n* = 20) was downloaded from GEO (GSE80672) aged 10, 18, 23, 27 months, with five mice at each age. For RegBC age prediction, genome was segmented and average methylation was calculated per region as per previous steps for either 5 kb RegBC or eps1000 DRegBC. And 5 kb RegBC, eps1000 DRegBC, Petkovich clock, and Meer clock were applied to CR data aged 10–27 months as per previous steps (see Section 4.4). The predicted age of CR data were compared to a control group (*n* = 74) which is C57 mice aged from 10 to 28 months sampled from the Petkovich blood data (training + crossvalidation test = 141). Age acceleration was then calculated as predicted age minus chronological age. ggboxplot (ggpubr version 0.5.0) was used to plot age acceleration. Wilcoxon test were used to compare the differences between the two groups. The median of the age acceleration was calculated in R and reported as the median error.

### 4.8 Annotation of regional blood clocks with published blood clock sites, GC counts and other metadata

Annotation files for the 5 kb RegBC and eps1000 DRegBC were initially created using SeqMonk and collated in Table S4. For example, to annotate the 5 kb RegBC with another clock, such as the Petkovich clock, the “Feature Probe Generator” in SeqMonk was used to generate probes based on each of the Petokvitch clock sites. “Bisulphite methylation over features” was then calculated (these values were not used, but quantitation was required to create an annotated probe report). An annotated probe report was created where each Petkovich site was annotated with an overlapping 5 kb RegBC window. In R, the results were collated to count the number of Petkovich CpGs that are present in each 5 kb RegBC region. These counts were then added to Table S4. This process was then repeated for the Meer clock. Similarly, overlapping eps1000 DRegBC regions and CpG islands (mm10 coordinates from Olova et al., 2018, originally from Illingworth et al., 2010) were mapped and processed in the same manner but instead of counts, names of overlapping regions were pasted directly into Table S4 for each 5 kb RegBC region. This entire process was repeated in the same way to annotate the eps1000 DRegBC. Total CGIs present in hyper- and hypomethylating regions were plotted using ggplot2.

Total base content for each RegBC probe was calculated with a custom perl script from https://github.com/NellyOlova/BS_bias (Olova et al., 2018). GC percentage per clock region was then calculated as total G bases plus C bases divided by region length. GC percentage for each eps1000 DRegBC region was then plotted using pheatmap (version 1.0.12) in R, ordered by methylation change (gradient) with age (calculated using the lm function in R). Methylation gradient was also plotted with pheatmap. Hyper- and hypomethylating regions were defined as any region with a positive or negative clock coefficient, respectively.

### 4.9 Annotating eps1000 DRegBC with ChromHMM labels and plotting log fold change

mm10 ChromHMM labels were downloaded from https://github.com/guifengwei/ChromHMM_mESC_mm10 (dense annotated bed file used) (Pintacuda et al., 2017). The bed file was loaded as an annotation track in Seqmonk. For our training data, probes were created based on the mESC bed file annotations using the “Feature Probe Generator” function. “Bisulphite methylation over features” was then calculated (these values were not used, but quantitation was required to create an annotated probe report). An annotated probe report was created, where the mESC ChromHMM probes were labelled with either overlapping 5 kb RegBC regions and eps1000 DRegBC regions. To calculate the number of base pairs each chromatin state was present within each eps1000 DRegBC region, the start site in bp for a given ChromHMM annotation was subtracted from the start site of the corresponding eps1000 DRegBC window. This gave the “starting flank”, i.e. the number of bp within the ChromHMM annotation a particular clock region starts. The “ending flank” of the same ChromHMM annotation/eps1000 DRegBC region was then calculated as the eps1000 region end coordinate subtracted from the ChromHMM end coordinate. Any starting or ending flanks that had values lower than 0 (i.e. negative values) were therefore the number of bp a ChromHMM annotation was not overlapping with a given DRegBC region. The total size of the eps1000 DRegBC region was then added to only negative value start and end flank numbers to give the total number of bp a given feature has within a given eps1000 DRegBC region. All remaining regions that did not have negative numbers added were then 100% contained within a given ChromHMM annotation. Any ChromHMM annotations that bordered with a clock region by only 1 bp were removed. The total bp annotation values within a eps1000 DRegBC region were then calculated as proportion within the total hyper- or hypomethylating region bp of the eps1000 DRegBC, i.e. each total feature bp in the hypermethylating regions were divided by the total number of bp of the hypermethylating clock regions and multiplied by 100 (the same procedure is done for hypomethylating annotations/regions). A list of 500 bp probes and eps1000 regions, both annotated with ChromHMM labels as well as calculated start/end flanks and bp overlap, can be found in Table S5.

To compare with approximate proportions of genomic chromatin states captured in RRBS, 500 bp probes were created for the Petkovich training data (CpGs < 5 reads filtered out) using the “running window generator” function in SeqMonk, with step size and probe size both set to 500 bp. Read count was quantitated and regions with 0 reads were removed (hence only regions with CpGs ≥ 5 reads were retained). These probes were then annotated with ChromHMM labels in SeqMonk in the same manner as for the RegBCs. Overlap between 500 bp probes and ChromHMM were calculated in the same manner as the eps1000 DRegBC regions (see previous paragraph). Proportion of genomic chromatin states was then calculated as the total bp for each chromatin state divided by the total bp of chromatin overlapping with the 500 bp probes (204,291,600 bp), multiplied by 100. Next, for eps1000 DRegBC regions and 500 bp genomic regions, “Repressed” and “Heterochromatin” annotations were merged and labeled as “Heterochromatin”, with bp of both annotations added together, given their similar properties. The proportion of chromatin states for either hyper- or hypomethylating regions were divided by the genomic proportion of the corresponding chromatin state, then log2 of the resulting calculation was plotted using ggplot2. Any missing values prior to calculation (i.e. if the DRegBC regions did not have a particular ChromHMM annotation) were substituted with 0. Log2 of 0 values resulted in “Inf” which was substituted with 0 for the purposes of plotting. Calculated proportions and log fold change can be found in Table S5.

## Supplementary Material

Figure S1

Table S1

Table S2

Table S3

Table S4

Table S5

## Data Availability

The data used in this study are all public domain and available from the following GEO repositories: GSE80672 at https://www.ncbi.nlm.nih.gov/geo/query/acc.cgi?acc=GSE80672. GSE120137 at https://www.ncbi.nlm.nih.gov/geo/query/acc.cgi?acc=GSE120137, GSE93957 at https://www.ncbi.nlm.nih.gov/geo/query/acc.cgi?acc=GSE93957. cgi?acc=GSE93957 and GSE121141 at https://www.ncbi.nlm.nih.gov/geo/query/acc.cgi?acc=GSE121141. Regions and weights that constitute the 5 kb RegBC and eps1000 DRegBC are available in Table S4 of this manuscript.
