# Supplementary figures and images for: Region-based epigenetic clock design improves RRBS-based age prediction
Source: Aging Cell. Author manuscript; Available in PMC 2023 Dec 13. (PMC10410054; doi:10.1111/acel.13866)

**A**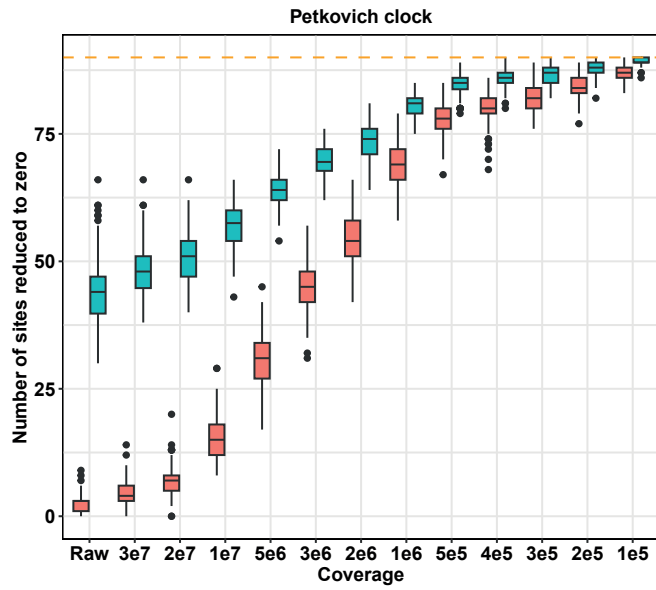**B**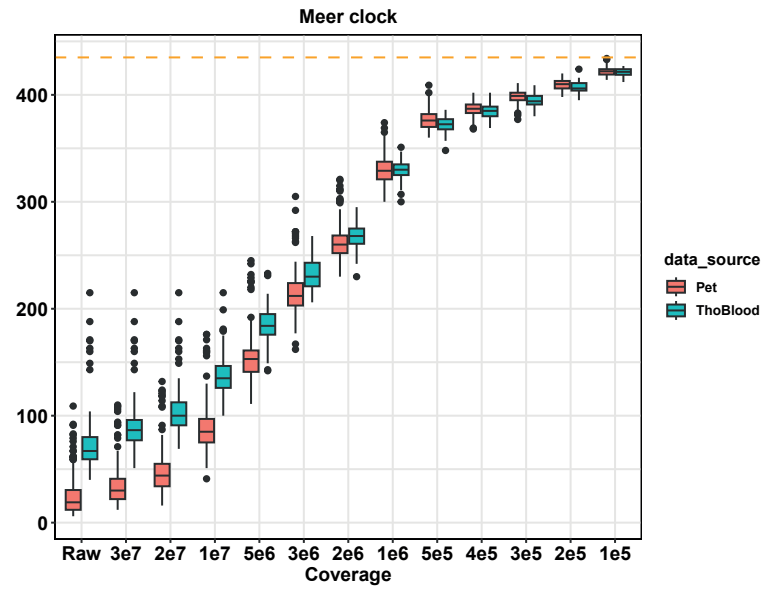**C**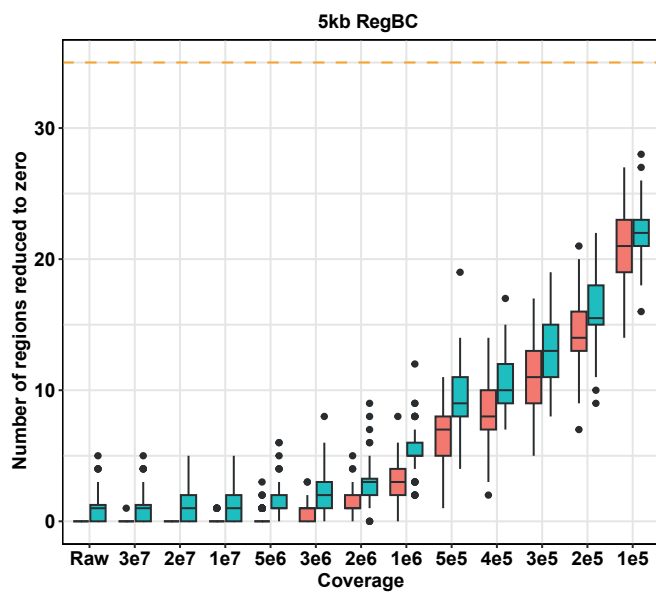**D**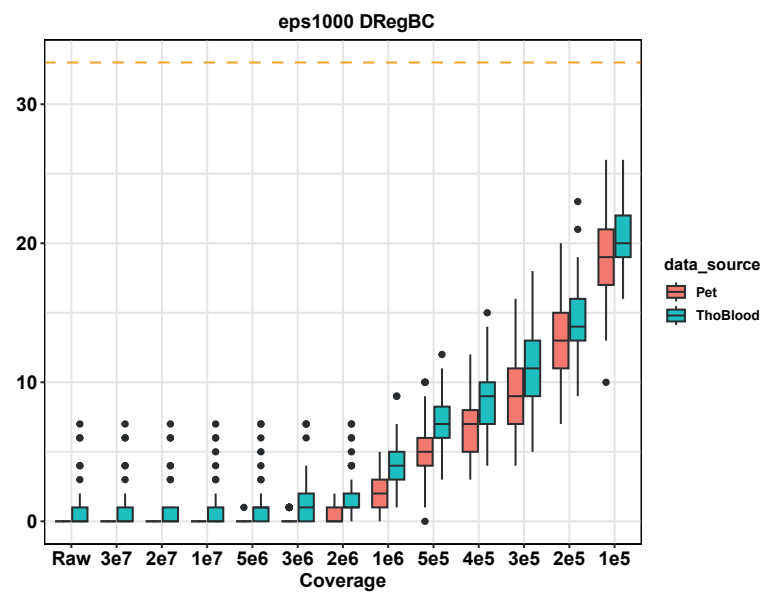

Supplement: Figure S1 [file EMS181574-supplement-Figure_S1.pdf]
